# 

**Published:** 1860-07

**Authors:** 


CONTENTS

OF THE

### Chicago Medical Journal for June, 1860.

ORIGINAL COMMUNICATIONS. PAGE.

ARTICLE 1.—Report of Surgical Cases treated at the City Hospital during the Spring Term, by Geo. K. Amerman, M. D., Attending Surgeon........ 377
ARTICLE 2.—Intercranial Tumor. By W. J. Moody, M. D........ 385
ARTICLE 3.—Stricture of the Urethra—Treatment of and operation for. By S. Wilkins, M. D. 389
ARTICLE 4.—Operation for Hernia. By F. B. Haller........ 393
ARTICLE 5.—G.adual Dislocation of the Femur, from Disease........ 394
ARTICLE 6.—Uterine Tumor—Déath—Autopsy........ 395
ARTICLE 7.—1, Case of Gun-shot Wound in the Chest—2, Poisoning by Laudanum. By A. Groesbeck, M. D........ 398
To the Medical Profession of Illinois........ 400

EDITORIAL.

Book and Pamphlet Notices........ 401 to 407
Percy on Reduction of Dislocation of the Hip, by Manipulation........ 408
The American Medical Association........ 409
" Physiological Incest"........ 410
Obituary........ 411
Items........ 412

SELECTIONS.

Mania a Potu........ 413
Hypophosphates of Soda and of Lime in the Treatment of Phthisis........ 417
Mr. Weeden Cooke, on the Treatment of Gonorrhœa and Gleet without Capabia........ 421
On the Treatment of Whooping Cough by Diluted Nitric Acid........ 424
M. Velpeau's Report on Coal-tar as a Disinfectant........ 426
Our Confreres of the Japanese Embassy........ 427
The Auricles of the Heart Act by their Elasticity and Contractility, not by Muscles........ 428
Comparative Value of Zinc in Chorea........ 429
Medical Students and Graduates in the United States during the Session of 1859-60........ 430
Bismuth in Gleet and Leucorrhœa........ 431
Iodized Glycerine in Skin Diseases........ 432
Remarks and Transalations illustrative of the Physiognomy of the Battle-field, and the Attitudes of the Dead........ 433
Practical Remarks on Fœtal Auscultation........ 438
Poisoning by Arsenic........ 439
Losses Sustained by Armies........ 440
Medical Students in Paris........ 440
Atropin in Epilepsy........ 440

